# Silent Hemoperitoneum Unveils Advanced Cancer

**DOI:** 10.7759/cureus.64494

**Published:** 2024-07-13

**Authors:** Cheng-Chieh Yen, Wan-Chen Chang, Chieh-Wei Chang

**Affiliations:** 1 Division of Nephrology, Department of Internal Medicine, Ditmanson Medical Foundation Chia-Yi Christian Hospital, Chiayi, TWN; 2 Peritoneal Dialysis Center, Ditmanson Medical Foundation Chia-Yi Christian Hospital, Chiayi, TWN; 3 Division of General Surgery, Department of Surgery, Ditmanson Medical Foundation Chia-Yi Christian Hospital, Chiayi, TWN

**Keywords:** hepatocellular carcinoma, segmentectomy, peritoneal dialysis (pd), end-stage kidney disease, bloody ascites

## Abstract

A 62-year-old male undergoing peritoneal dialysis (PD) for over two years presented with sudden bloody peritoneal dialysate, but no other symptoms. Laboratory tests indicated anemia, and a computed tomographic scan revealed a 4.4 cm tumor in the liver with hemoperitoneum, leading to a diagnosis of ruptured hepatocellular carcinoma (HCC), stage IIIB T4N0M0. The patient underwent a successful laparoscopic segmentectomy, and PD was resumed after a month of hemodialysis without complications. This case underscores the importance of considering malignancy in PD patients presenting with hemoperitoneum, as timely detection of HCC can significantly improve prognosis.

## Introduction

Peritoneal dialysis (PD) is a widely used modality for treating patients with end-stage renal disease (ESRD). It involves the infusion of dialysate into the peritoneal cavity, which facilitates the removal of metabolic waste and excess fluid via the peritoneum. PD offers comparable survival benefits to hemodialysis [1] while providing greater lifestyle flexibility and improved quality of life for patients [2]. However, its utilization can be hindered by complications, such as infection, catheter dysfunction, and ultrafiltration failure [3]. Hemoperitoneum is a common complication observed in PD within routine clinical practice, identified by the presence of more than 2 mL of blood in 1 L of dialysate. The etiology of hemoperitoneum may be attributed to various factors, including PD catheter, intra-abdominal and pelvic organs, vasculature, infections, or iatrogenic interventions [4]. Herein, we present a case of a patient undergoing PD who experienced an episode of incidental hemoperitoneum.

## Case presentation

**Figure 1:**
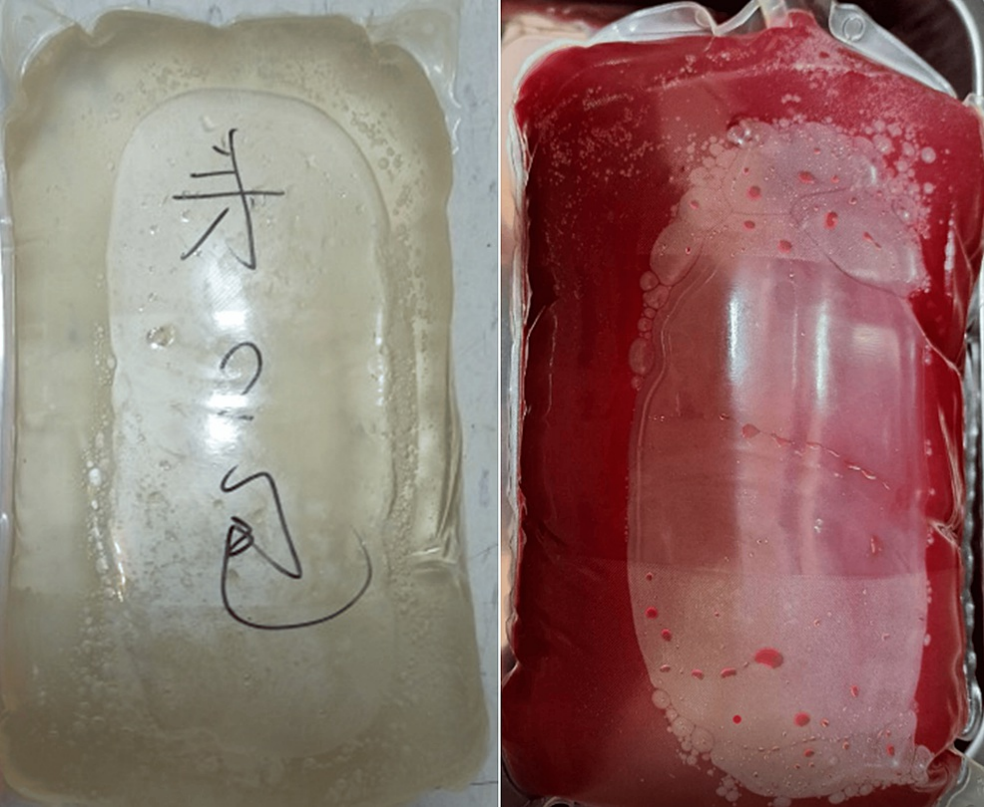
The appearance of dialysate exhibited clarity and translucency (left) until the occurrence of this episode, where it exhibited bloody and opaque (right).

A 62-year-old male patient with a body mass index of 26.8 kg/m² and medical history of hypertensive cardiovascular disease, coronary artery disease, and dyslipidemia has been on automated PD for over two years due to ESRD secondary to chronic glomerulonephritis. The patient reported no history of diabetes mellitus or alcohol consumption, and regular examinations revealed no hepatitis infection. The PD process had been consistently stable until an abrupt onset of bloody peritoneal dialysate drainage was observed (Figure 1). The patient denied recent trauma, medical interventions, or adjustments to medication. There were no reported symptoms, including fever, chills, turbid dialysate drainage, abdominal pain, nausea, vomiting, or alterations in bowel habits. Consequently, he sought assistance at our emergency department (ED).

**Figure 2:**
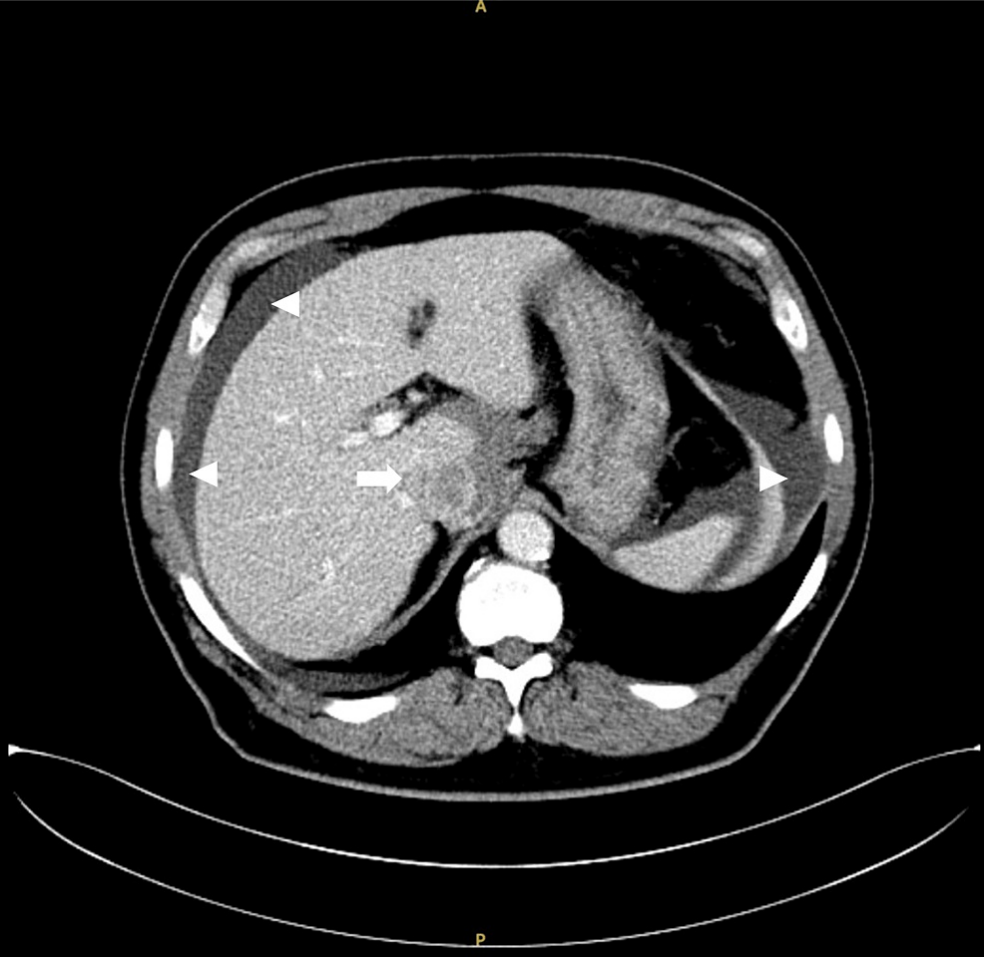
A 4.4 cm heterogeneous tumor (arrow) was at the caudate lobe of the liver, accompanied by hemoperitoneum (arrowheads).

The patient exhibited hypertension without signs of hyperthermia, tachycardia, or tachypnea at ED. Laboratory data indicated anemia without leukocytosis. Analysis of the dialysate revealed a red blood cell count of 113,000/μL with hematocrit of 1.6% (Table 1). Computed tomography (CT) was conducted to investigate hemoperitoneum, revealing a 4.4 cm heterogenous tumor located at the caudate lobe of the liver, accompanied by hemoperitoneum (Figure 2). The interventional radiologist was consulted due to suspicion of tumor rupture, and subsequently, the patient was admitted for further management.

**Table 1:** Laboratory data at the emergency department

Item (unit)	Value	Reference range
Serum		
Leukocyte (10^3^/μL)	7.07	3.5–9.9
Hemoglobin (g/dL)	9.0	14–18
Platelet (10^3^/μL)	178	130–400
Peritoneal effluent		
Hematocrit (%)	1.6	-
Total protein (g/dL)	0.3	-
Red blood cell count (1/μL)	113000	-
White blood cell count (1/μL)	60	-
Neutrophil (%)	30	-
Lymphocyte (%)	65	-
Monocyte (%)	1	-
Eosinophil (%)	4	-
Basophil (%)	0	-

**Figure 3:**
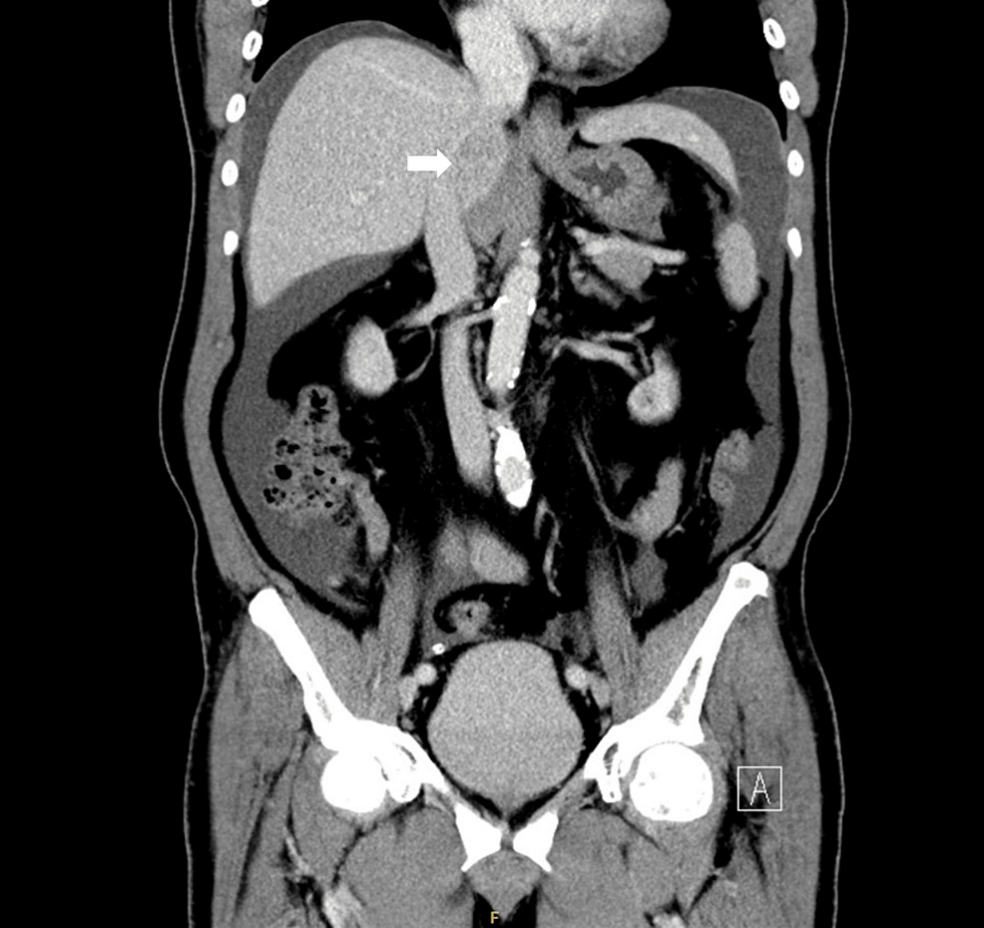
The tumor featured evidence of portal washout (arrow).

Upon admission, enhanced CT revealed a hepatic tumor exhibiting portal washout, strongly suggestive of ruptured hepatocellular carcinoma (HCC) (Figure 3). There was no evidence of extrahepatic metastasis, multiple tumors, main bile duct involvement, or tumor thrombus in the main portal vein or vena cava. Based on the American Joint Committee on Cancer Staging criteria, the patient was diagnosed with stage IIIB T4N0M0 HCC. Laboratory data indicated normal liver function tests and tumor marker levels (Table 2). Given the reduced size of the tumor post-rupture, an extended left hepatectomy was considered to involve an excessive loss of liver volume. Consequently, the patient underwent an isolated laparoscopic S1 segmentectomy. Histological analysis of the resected specimen confirmed the diagnosis of HCC. Hemodialysis was initiated to allow peritoneal rest for one month; thereafter, PD was resumed without complications. Chemotherapy was recommended due to the advanced stage of his HCC; however, the patient declined this treatment option. Fortunately, follow-up CT scans obtained two months later showed no signs of recurrence. The patient continues to be monitored through regular outpatient follow-up.

**Table 2:** Laboratory data upon admission

Item (unit)	Value	Reference range
International normalized ratio	0.98	0.85–1.15
Activated partial thromboplastin time ratio	1.06	-
Glutamic oxaloacetic transaminase (U/L)	20	8–38
Glutamic pyruvic transaminase (U/L)	19	4–44
Total bilirubin (mg/dL)	0.50	0.2–1.2
Direct bilirubin (mg/dL)	0.14	0–0.4
Albumin (g/dL)	3.8	3.8–5.3
Alkaline phosphatase (U/L)	176	104–338
Ammonia	38	12–66
α-fetoprotein (ng/mL)	4.2	0–20
Carcinoembryonic antigen (ng/mL)	3.1	0–5
Carbohydrate antigen 19-9 (IU/mL)	6.8	0–37

## Discussion

Stengel's analysis of dialysis patients in the United States, Europe, Australia, and New Zealand revealed an increased malignancy occurrence rate of 10-80% [5]. Lee et al. reported higher risks of HCC, genitourinary tract cancers, and thyroid cancers in dialysis patients, with no significant difference between those undergoing PD and hemodialysis [6]. In addition, Fabrizi et al. demonstrated that HCC significantly impairs survival in dialysis patients [7]. The elevated incidence and prevalence of HCC in ESRD patients can be attributed to various patient-related factors, including lifestyle choices, substance use, and environmental exposures. Disease-related factors, including genetic conditions, inflammation, comorbidities, and infection, also contribute to this increase. In addition, treatment-related factors including the use of erythropoietin-stimulating agents and immunosuppressants play a role in this complex scenario [8]. Among the ESRD cohort, the diagnosis of HCC commonly experiences delays compared to non-ESRD cohorts, thereby contributing to an increased mortality rate subsequent to diagnosis [9]. Timely identification of HCC holds paramount significance for the overall prognosis of patients undergoing PD.

Our patient did not exhibit any gastrointestinal or jaundice symptoms, which could potentially be attributed to the deep-seated location of the malignancy. In addition, regular sonographic follow-up was not conducted due to the absence of hepatitis infection. The discovery of advanced HCC ensued from the investigation of hemoperitoneum, with potential hepatic origins attributed to traumas, tumors, or cysts [4]. The manifestation of hemoperitoneum as the sole symptom may serve as a potential indicator, providing an opportunity for early detection of malignancy and leading to an improved prognosis in the PD cohort.

## Conclusions

This case shows that hemoperitoneum in PD patients can indicate serious conditions like HCC. The timely investigation of bloody dialysate led to the successful diagnosis and treatment of advanced HCC, even without typical symptoms or risk factors. Regular monitoring in PD patients is essential for early detection and better outcomes.
